# Domestic dogs (*Canis familiaris*) recognise meaningful content in monotonous streams of read speech

**DOI:** 10.1007/s10071-025-01948-z

**Published:** 2025-04-12

**Authors:** Holly Root-Gutteridge, Anna Korzeniowska, Victoria Ratcliffe, David Reby

**Affiliations:** 1 School of Psychology, University of Sussex, Falmer, East Sussex, UK; 2 School of Life and Environmental Sciences, University of Lincoln, Lincoln, Lincolnshire, UK; 3 Defence Science and Technology Laboratory, Salisbury, Wiltshire, UK; 4 ENES Bioacoustics Research Lab / Lyon Neuroscience Research Centre (CRNL), University of Saint-Etienne, CNRS UMR5292, INSERM UMR_S 1028, Saint-Etienne, France; 5 Institut Universitaire de France, Paris, France

**Keywords:** Dog-directed speech, Heterospecific communication, Speech recognition, Word recognition, Human-animal communication

## Abstract

Domestic dogs (*Canis familiaris*) can recognize basic phonemic information from human speech and respond to commands. Commands are typically presented in isolation with exaggerated prosody known as dog-directed speech (DDS) register. Here, we investigate whether dogs can spontaneously identify meaningful phonemic content in a stream of putatively irrelevant speech spoken in monotonous prosody, without congruent prosodic cues. To test this ability, dogs were played recordings of their owners reading a meaningless text which included a short meaningful or meaningless phrase, either read with unchanged reading prosody or with an exaggerated DDS prosody. We measured the occurrence and duration of dogs’ gaze at their owners. We found that, while dogs were more likely to detect and respond to inclusions that contained meaningful phrases spoken with DDS prosody, they were still able to detect these meaningful inclusions spoken in a neutral reading prosody. Dogs detected and responded to meaningless control phrases in DDS as frequently as to meaningful content in neutral reading prosody, but less often than to meaningful content in DDS. This suggests that, while DDS prosody facilitates the detection of meaningful content in human speech by capturing dogs’ attention, dogs are nevertheless capable of spontaneously recognizing meaningful phonemic content within an unexaggerated stream of speech.

**Supplementary Information:**

The online version contains supplementary material available at 10.1007/s10071-025-01948-z.

## Introduction

After more than 14,000 years of domestication, domestic dogs (*Canis familiaris*) have a close and well-developed relationship with humans (Vilà et al. 1997; Thalmann et al. 2013), making them an excellent model for exploring human-animal communication. Dogs are highly attentive to human speech (Kaminski et al. 2004; Fukuzawa et al. 2005; Adachi et al. 2007; Pilley and Reid 2011; Pilley 2013; Gibson et al. 2014; Ratcliffe et al. 2014; Ratcliffe and Reby 2014; Root-Gutteridge et al. 2019; Boros et al. 2020) and respond to both segmental phonemic cues (Baru 1975 (problematic - welfare validity concerns); Fukuzawa et al. 2005; Ratcliffe and Reby 2014) and emotional prosodic cues (Scheider et al. 2011; Ratcliffe and Reby 2014). Furthermore, dogs can learn to recognise human voices (Adachi et al. 2007; Root-Gutteridge et al. 2019), commands (Mills 2005), and even referential words (Kaminski et al. 2004; Pilley and Reid 2011; Dror et al. 2021; Fugazza et al. 2021). While some of these abilities may use the same acoustic features as when discriminating between conspecific vocalisations (Yin and McCowan 2004; Maros et al. 2008; Taylor et al. 2009; Péter et al. 2014), the extent to which dogs are capable of extracting segmental information that is unique to human speech remains poorly understood. Here, to fill this gap, we explore dogs’ ability to identify speech that is meaningful to them among a stream of read speech that is meaningless to them, while also testing the effects of speaker sex and reading prosody on their performance.

When addressing dogs, humans often use a speech register called dog-directed speech (DDS) which, like infant-directed speech (IDS), is characterized by increased intonation and pitch range, and is thought to attract the listener’s attention (Mitchell and Edmonson 1999; Xu et al. 2013; Gergely et al. 2017; Ben-Aderet et al. 2017; Lesch et al. 2019) and make word recognition easier (Thiessen et al. 2005). There is some evidence that adult dogs prefer to associate with people who produce DDS compared to adult-directed speech (Braem and Mills 2010; Jeannin et al. 2017; Ben-Aderet et al. 2017; Andics and Miklósi 2018; Benjamin and Slocombe 2018). Male and female owners are known to exhibit differences in their use and presentation of DDS, with male owners producing less exaggerated speech compared to female owners (Prato-Previde et al. 2006). Furthermore, while dogs respond differently to male and female voices (Ratcliffe et al. 2014; Gergely et al. 2017), the effect of speaker sex on word recognition has not been investigated yet and is thus included in our experimental design. (Prichard et al. 2018; Gábor et al. 2020) Dogs may follow commands more efficiently when presented in DDS (Mills et al. 2005). Yet the specific contribution of speech prosody to dogs’ perception of speech signals is not well understood, and, in particular, it remains to be established whether DDS prosody is essential for command recognition. To test this, we included prosody type as a condition in our experimental design.

Speech is typically presented as a long stream of phonemes, often against background noise provided by either environmental sound or other speakers. A subset of human speech research has examined the “cocktail party effect”, first identified in humans by Cherry (1953), where speech of interest is extracted from background “babble”, and attended to while the “babble” is discarded (Gábor et al. 2020). Humans can separate these streams and pick out the salient speech stream against multi-talker babble (Cherry 1953). There is evidence that dogs are capable of recognising their name when presented in a multi-speaker babble, even outperforming one-year-old human infants, provided the loudness of meaningless-to-them speech and their own name were of equal intensity (Mills et al. 2005). The dogs were shown to attend to their names by orienting towards the sound source (Mallikarjun et al. 2019). This suggests that they have some ability to recognise familiar content when set in noise.

Similar to the ability to separate speech from noise, language comprehension also requires the ability to parse streams of speech into meaningful phonemic units, a process known as speech segmentation (Thiessen et al. 2005). Because speech segmentation is a key part of language acquisition (Thiessen et al. 2005), it has received much scrutiny in humans (Jusczyk et al. 1999; Mattys et al. 1999; Thiessen et al. 2005; Bortfeld et al. 2005; Seidl and Johnson 2006; Bergelson and Swingley 2012; Vihman 2017; Westermann and Mani 2017). Human infants can perform this speech or word segmentation from birth (Mandel et al. 1995; Fló et al. 2019), and by 6 months, they can recognise target words if their name precedes them (Bortfeld et al. 2005). Infant-directed speech is used to emphasise word boundaries through exaggerated enunciation and increase attentiveness through increased modulation of tone, which may aid the comprehension of speech and emphasise word boundaries, characteristics also seen in dog-directed speech (Burnham and Francis 1998). The ability to segment speech partially depends on statistical learning of the probability that certain sequences of phonemes are more likely than others, allowing the prediction of word boundaries. For example, Fló et al. (2019) claim that *bana*… is likely to be followed by “*na*”, but “*banana*” can be followed by “split, peel, republic”, and thus the parsing of the sequence beyond the trisyllabic word is more difficult. Therefore, listeners are more likely to succeed at parsing complex utterances if the speech presented is familiar and the phonemes follow known statistical distributions.

Beyond statistics, exaggerated prosody, which can emphasise pauses and breaks between words, can give a further cue to speech segmentation. Floccia et al. (2016) found that young infants’ ability to segment speech depended on the use of exaggerated, infant-directed speech prosody, and discussed the impact of prior experience of speech prosody on word recognition. However, Fló et al. (2019) determined that even newborn humans do not require prosodic cues to recognise words. Previous research has shown that speech with flat fundamental frequency is perceived by human listeners to be less intelligible than more modulated speech (Brokx and Nooteboom 1982; Laures and Weismer 1999; Binns and Culling 2007). While some studies have found that prosodic cues are less useful to adult listeners learning new languages than to infants (Gerken 1996; Matzinger et al. 2021), this finding was not upheld in other studies where prosody was found to be important to adult speech segmentation and learning (Valian and Levitt 1996; Kim et al. 2012; Spring et al. 2013). Overall, learning to segment speech is supported by both exaggerating speech prosody and the pauses between words, but these pauses are rare in adult-directed speech (Matzinger et al. 2021). Other factors influencing speech perception include the amplitude of speech, the speaker’s familiarity, and their sex (Cherry 1953; Fant and Tatham 1975; Kuhl 1988; Childers and Wu 1991; Nygaard and Pisoni 1998; Binns and Culling 2007; Johnson 2008). Thus, it can be predicted that familiar speech presented with highly modulated frequency and increased prosody will gain more attention and be easier to segment from streams than more monotonous speech, but that segmentation of monotonous speech should still occur. Here, we examine whether brief speech pauses are sufficient for dogs to parse familiar, meaningful content from unfamiliar speech streams.

Dogs constitute an excellent model for comparative research into speech perception and heterospecific listening due to their near-constant exposure to human speech and their own interest in following our cues (Taylor et al. 2014). Dogs are commonly exposed to complex speech and dog owners embed meaningful phrases in longer, non-salient speech streams when addressing their pets, suggesting that they expect that their dogs are capable of speech-recognition (Mitchell and Edmonson 1999). Indeed, dogs appear to possess the neural architecture to support it(Boros et al. 2021) and exhibit different processing responses when hearing either familiar or unfamiliar words, supporting the idea that they learn words, rather than just intonational cues or physical gestures (Prichard et al. 2018; Gábor et al. 2020). However, it has been shown that dogs’ ability to recognise words falls when ambiguity increases, such as when commands are embedded in more complex sentences or following pauses (Braem and Mills 2010), and it is not clear how far the speech register affects their ability to detect (or their propensity to respond) to meaningful words. Researchers used a combination of fMRI to investigate dog brain structure and EEG scans to investigate whether dogs have the neural mechanisms required to parse speech (Boros et al. 2021). They familiarised the dogs with a stream of artificial speech, with different frequencies of word repetition and paired words and then tested the dogs’ event-related responses (ERPs) to the presentation of the learnt speech (Boros et al. 2021). They found that the dogs showed stronger responses to the high frequency artificial words than the low-frequency artificial words, suggesting that dogs segmented the speech into the known syllables and were capable of statistical learning of the co-occurrence of speech syllables (Boros et al. 2021). However, it remains to be established whether dogs express this ability when processing normal speech and in their normal, everyday behaviour.

Dogs produce sequences of barks which can be separated by pauses or continuous, “fused” barks which overlap (Yin and McCowan 2004). However, understanding which units of these sequences are meaningful and decoding them is challenging as it requires pairing call with context (Yin and McCowan 2004), which can be challenging to determine. However, there is evidence from dingoes (*Canis familiaris dingo*) that multi-segment syllables are combined to form novel meanings (Deaúx et al. 2016). However, it is difficult to establish how far dogs parse the sequences of barks or respond to familiar content within sequences. Using human speech overcomes this issue as it is already established which the meaningful units are and what the response should be. Furthermore, it has been suggested that domestication has shaped the speech perception abilities of species (Hare et al. 2002), thus they may be more strongly pronounced in the species which are accustomed to hearing human speech, including dogs. We suggest that dogs are a good model for exploring how well animals recognise call units within sequences as they produce call sequences and have had long-term exposure to human speech as meaningful. Here, we explore whether dogs are able to discern the presentation of meaningful content in a stream of meaningless-to-them speech and therefore their ability to parse sequences to identify salient phrases.

In order to investigate whether dogs can detect meaningful speech phrases embedded in a stream of speech, and how important tone cues are to their speech recognition we tested dogs’ ability to recognise a meaningful phrase (“[dog’s name], come on then”) within a longer speech stream, presented in either DDS speech or neutral-tone speech. We assessed whether dogs exhibited attention to their owner on hearing the salient content in (a) DDS and (b) neutral reading prosody (NRP) voice speech. We predicted that while dogs may be able to respond to speech either containing meaningful content or presenting DDS prosody, responses should be strongest to speech combining meaningful content pronounced using dog-directed speech. Finally, we investigated whether dogs would either respond more readily to NRP presented by male owners as compared to female owners or show less differentiated responses to men’s DDS and NRP speech.

## Materials and methods

To test whether dogs would respond more to meaningful than non-meaningful content pronounced in the same speech register, we first piloted their response to speech in Dog-Directed Speech register (DDS, Pilot Experiment), then tested their response to their owners’ speech in Neutral Reading Prosody (NRP) vs. DDS (Experiment 1: Prosody), and explored whether owner sex modulated the response of dogs in these tasks (Experiment 2: Sex).

### Stimuli

70 owners were recorded reading aloud one of three short (15–20 s) passages from the standard oral reading passage “the rainbow passage” (Fairbanks 1960), with the test phrases produced after 7–12 s as part of the text. The non-meaningful (control) phrases were “[Alfie / Bertie], pass me a coffee!” and the meaningful phrase was “[Dog’s name], come on then!”, chosen as these words had the highest frequency of use by English-speaking owners during interactions with their dogs and were therefore likely to be meaningful to all dogs (Mitchell and Edmonson 1999). The duration of the target phrases was between 0.7s and 2.5s (mean = 1.4s, std. dev. = 0.2), depending on the speaker’s natural talking speed and the number of syllables in the dog’s name. In total, three different extracts of the same length were used and the phrases were included within the sentences, i.e., “There is, according to legend, a boiling pot of gold at one end. People look, but no one ever finds it. When a man looks for something beyond his reach, his friends say, *[Bertie*, *pass me a coffee] / [Dog’s name*, *come on then]*, he is looking for the pot of gold at the end of the rainbow. Throughout the centuries people have explained the rainbow in various ways.” (See ESM for extracts 2 and 3.) The time it took the owners to reach the included phrase depended on the speed of their natural speech (mean = 8.7s, std. dev. = 1.2s) but was consistent across readings by the same individual.

The choice of extract was randomised but if the dog had a name too similar to Alfie or Bertie, they were given an abstract that presented the non-similar name (e.g., the participant dogs Betty and Beans heard the extract which contained Alfie, not Bertie as the control name). For each dog, the same extract was used for all conditions. Voice recordings were made on a Zoom H4N-Pro handheld recorder (Zoom) in a sound-proof booth on campus at University of Sussex. To avoid habituating the dogs to the speech, owners were recorded reading the passages without the dog present in the recording booth and were asked to imagine they were speaking to the dog. Owners were asked to produce the target phrases in (a) their normal reading voice prosody (NRP) and (b) dog-directed speech prosody (DDS). There was an expectation that the DDS speech would show increased pitch and range compared to NRP and that this would be more interesting to the dogs (Lesch et al. 2019). Thus, two recordings were made for the Pilot Experiment: DDS-meaningful and DDS-control; four recordings were created by each owner for the main experiments: NRP-meaningful, NRP-control, DDS-meaningful, and DDS-control.

All the voice recordings were cut and aligned using the sound software Audacity (Mazzoni and Dannenberg 2015) and the amplitude normalized to -9dB. Mean and coefficient of variation of fundamental frequency (*f*_o_CV = (*f*_o_ standard deviation / *f*_o_ mean) *100) were measured in Praat (Boersma and Weenink 2009). *f*_o_CV provides a standardised measure of *f*_o_ variability independent of *f*_o_ height that takes perception into account (i.e., a modulation of 10 Hz around 100 Hz is perceptually equivalent to a modulation of 100 Hz around 1,000 Hz). Values are presented in Table 1. Within sexes, mean *f*_o_ differed significantly between target phrases (female: F_3,108_ = 68.3, *p* < 0.001; male: F_3,43_ = 43.0, *p* < 0.001). However, mean *f*_o_ did not differ significantly for control vs. meaningful within DDS and NRP registers (LMM: *p* > 0.05 for all), but DDS and NRP phrases did differ significantly from each other (*p* < 0.001 for all). For male owners, coefficient of variation did not differ between any comparison (*p* > 0.25 for all comparisons, F_3,43_ = 0.6, *p* = 0.592 overall). For female owners, coefficient of variation did differ significantly overall (F_3,127_ = 5.1, *p* = 0.002) but only for DDS-Meaningful to all others (*p* < 0.010 for all DDS-meaningful pairwise comparisons), while other pairwise comparisons were non-significant at *p* > 0.2 for all. Thus, DDS speech differed to NRP speech but phrases within speech registers were not significantly different except for female coefficient of variation.

Linear mixed models were applied to a subsample of (a) 5 female and (b) 5 male voices and were used to confirm that the mean fundamental frequency and coefficient of variation of the included phrases presented in NRP did not differ significantly from that of the rest of the read speech. (Mean fundamental frequency LMMs: female - F_1,14_ = 2.9, *p* = 0.108; male– F_1,14_ = 0.6, *p* = 0.449. Coefficient of variation LMMs: female - F_1,14_ = 2.4, *p* = 0.141; male– F_1,14_ = 2.9, *p* = 0.111.)

**Table 1:** Mean and standard deviation of mean and coefficient of variation of fundamental frequency for (a) the target phrases produced by all speakers and (b) the target phrase and entire speech of 10 speakers

Sampled speech	Speech	Mean f_o_ (Hz)	Coefficient of variation
All speakers’ target phrases across all conditions	DDS-meaningful	375 +/-139	14 +/-8
	DDS-control	346 +/-146	16 +/-6
	NRP-meaningful	158 +/-36	12 +/-4
	NRP-control	162 +/-34	13 +/-5
10 speakers’ NRP speech for target phrase vs. read speech	NRP-target phrase	153 +/-38	15 +/-5
	NRP-background speech	159 +/-40	14 +/-4

### Participants

Fifty-three privately-owned dogs were recruited through Facebook adverts, flyers, and personal contacts, and tested in a designated testing room on campus at University of Sussex. A total of 57 owners (17 male, 40 female) participated, with a maximum of 3 dogs per owner. (As experiment 2 examined the effect of owner sex, the dogs’ response to both their male and female owners was tested, leading to more owners than dogs.) Trials were discarded if the dog was distracted by non-stimuli sounds or events, e.g., background noise (*n* = 1), the dog was barking continuously (*n* = 1), or if they moved out of camera shot (*n* = 2). We retained data from 49 dogs (24 females and 25 males), from 39 breeds and cross-breeds, aged between 9 months and 12 years old (mean = 4.1 years, SD = 2.9 years) in our analyses (see ESM Table 1 for details following Volsche et al.’s (2023) suggested format).

### Protocol

Dogs were introduced to the room and given up to 20 min to freely explore and habituate to the space. Trials began once the dog was considered to be relaxed based on the owner’s assessment and the dog’s behaviour, e.g., the dog adopted a rest posture such as a sit, they were not panting, barking, whining, or attempting to access the owner. No dogs appeared to be stressed either before, during, or after the trials, using the signs of stress first marked by Beerda et al. (1997), e.g., panting, whining, or circling.

**Fig. 1:**
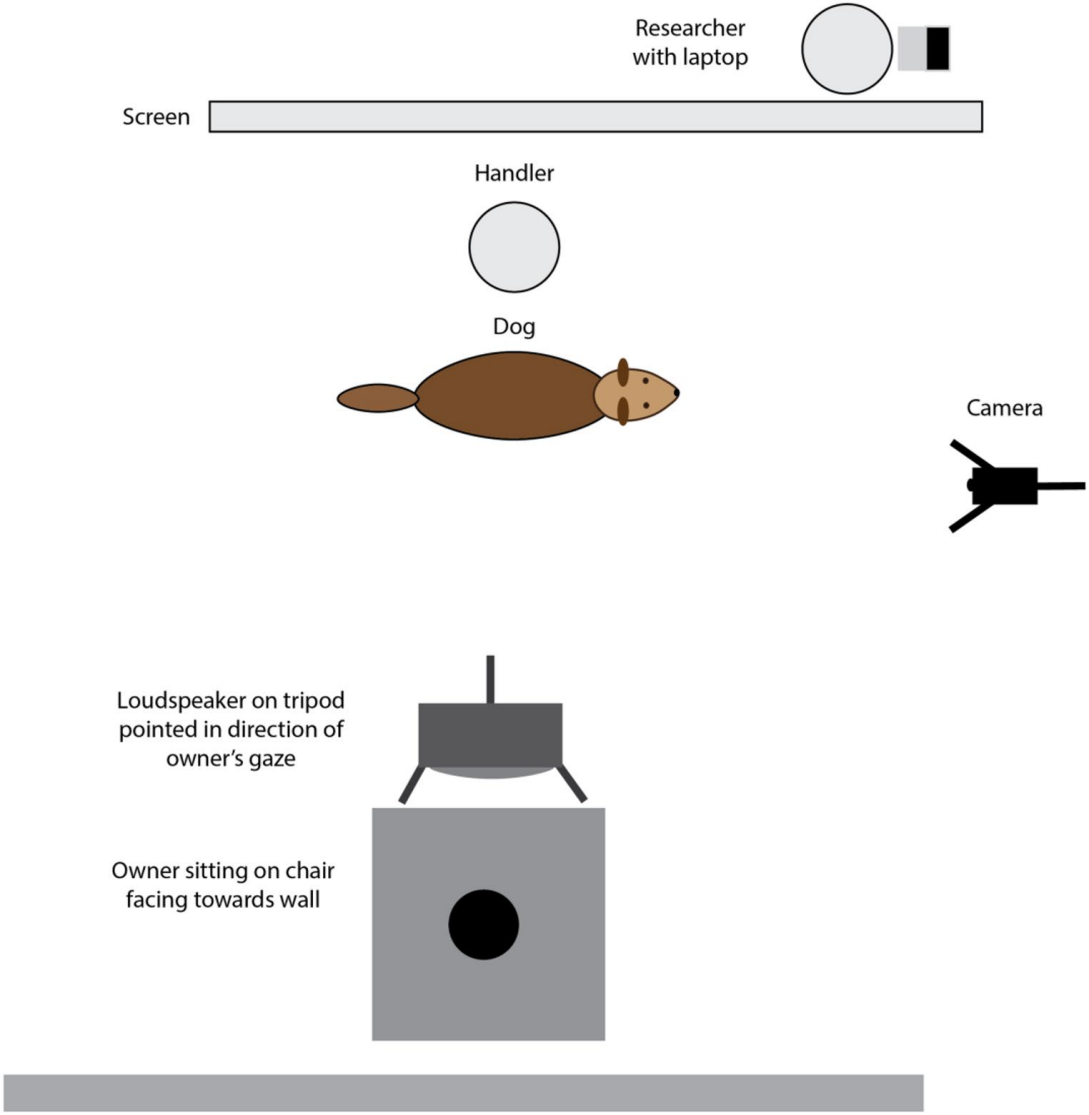
Experimental set-up in testing room at University of Sussex with the speaker positioned to the dog’s left. In half of the trials, this arrangement was reversed with the speaker positioned to the dog’s right. The owner was seated facing away from the dog wearing headphones and listening to music while the dog was positioned behind their chair and held on a loose lead by a handler. The speaker was positioned behind the owner’s head to simulate them speaking

During all trials, the owners wore noise-cancelling headphones (TaoTronics TT BH-047) and listened to music while seated in a chair at 90 degrees to the dog (Fig. 1), with their back to the dog and instructed not to turn to look at the dog. A single Behringer Europort MPA40BT-PRO speaker was set on a tripod behind the owner’s head and set to conversational volume (approx. 65dB measured at the dog’s position). The experimenter stood out of the dog’s sight line and controlled the stimuli from an Apple MacBook Pro. The dogs were held on a loose lead by the handler and allowed some freedom of movement. While the handler was consistently one of two researchers, their familiarity to the dog could vary from “completely unfamiliar” to “person the dog met on more than one occasion but do not have a close relationship to” if the dog had participated in a previous study before or belonged to a friend of the researchers.

The dogs were positioned either to the left or the right of the speaker, and this position was counter-balanced across dogs within experiments, with half to the left and half to the right. The dogs’ reactions were filmed on a Sony FDR-AX100 camcorder (Sony) on a tripod positioned approximately 1.5–2 m from the dogs’ starting position. The inter-trial interval depended on the dogs’ disposition. If the dog was calm, e.g., resting in one position, such as lying down, and not vocalising or attempting to attract the owner’s attention, the trial interval was less than 2 min. However, if the dog was restless or distracted, e.g., roaming around the room, vocalising, or focusing intently on sounds or scents in the room, a short break of a few minutes was provided, and the dog was sometimes taken out of the room and returned.

As some owners brought more than one dog and some dogs heard more than one owner, we considered each pairing of owner and dog to be a unique dyad, and thus the unit of comparison was dyad not owner or dog.

Whether the dogs gazed at their owner or not in the 10s period following the included phrase was used as the broadest metric of attention, while duration of gaze was used as the index of attention. None of the dogs were looking at or fully oriented towards their owner immediately prior to the onset of the target phrase, which would have been a criterion for dropping the trial. The trial ended 10s after the onset of the included phrase. Throughout the trials, the handler stood still if the dog was still or followed them if they approached the owner after target phrase (no dog approached before the target phrase).

### Pilot experiment: the effect of meaning on dogs’ responses to content presented in dog-directed speech (DDS) prosody

The pilot experiment was designed to test whether dogs responded differently to inclusions containing meaningful phrases vs. meaningless, control phrases, in both cases spoken with dog directed prosody (DDS). If they did not respond to the DDS presentation of speech, it was felt that it was unlikely that they would do so to NRP speech and that a new protocol would be required. Twenty-two dogs were tested, and 40 trials from 20 dogs were retained, with 2 dogs removed because they moved out of camera view during the stimulus. All owners included in this experiment were female. Each dog was presented with a recording of their female owner reading the text twice, once including the meaningful phrase and once including the control phrase. The order of presentation of meaningful and control phrase recordings was counter-balanced across dogs.

### Experiment 1 prosody: impacts of prosody and content on response

To better explore the effects of prosody and content, the pilot protocol was repeated with a total of 43 owner-dog dyads and all four speech conditions, adding NRP-meaningful and NRP-control to the DDS versions. The dogs heard all four speech conditions in pseudo-randomised presentation, counter-balanced across dogs. A total of 172 trials were retained (13 dogs heard 8 trials, with 4 trials from their male owner and 4 trials from their female owner, but one of these dogs moved out of shot).

### Experiment 2 sex: the effects of sex on dogs’ responses to content and prosody

During initial data collection, it was noted that some of the dogs appeared to be more responsive to the male owner’s NRP speech than their female owner’s NRP speech. Therefore, we decided to explore the potential effects of speaker sex on their responses. Thus, we tested whether dogs hearing both their male and female owners would respond differently to them across all four conditions of meaning and prosody, with an expectation that NRP from male owners could elicit more or stronger responses than female NRP due to the smaller differences between male NRP and DDS.

Each of the 13 dogs heard a total of 8 trials, 4 from each owner. To avoid the possible effect of learning on response to the target phrases, as the same text passage was used throughout, the NRP trials were always played first for each owner, with control and meaningful phrase presentation cross-balanced within DDS conditions. Both owners were present in the room, but the non-participant (e.g., the male while the female was “talking” to the dog) was kept out of view to prevent any “clever Hans” effect influencing the results.

One dog was removed from the dataset because he moved out of camera shot while reacting to his owners’ voices and thus his responses could not be coded. Another dog (Emma, terrier) had been previously tested in the pilot experiment with a gap of several months between tests, but all other dogs experienced the stimuli as a novel presentation and it was expected that Emma would not retain her memories of the pilot experiment or be primed by them. (Most studies of memory in dogs focus on timespans measured in minutes (Fiset et al. 2003; Fugazza et al. 2016), but there is some evidence they can retain memories for a year or more for trained tasks (Lazarowski et al. 2021), but this does not address non-reinforced experience. However, we note that Emma may represent an outlier in the data.) Thus, 96 trials were retained from 12 dogs in total, with each dog hearing a total of 8 trials, including all four speech presentations from both their male and female owners.

All eight trials were performed on the same day and between trial intervals varied from a few minutes to more than 20 min depending on the behaviour of the dog, e.g., engagement in other activities like sniffing or investigating the area. We counterbalanced the presentation of male and female owners’ speech, but each dog heard all four trials from each owner as a block which was not divided (e.g., male owner trials x 4 then female owner trials x 4, but not male owner x 2 then female x 2 etc.). The dogs heard the same order of presentation for both male and female owners (e.g., 1) NRP-meaningful, 2) NRP-control, 3) DDS-meaningful, then 4) DDS-control for both owners, cross-balanced order across dogs) to avoid order effects on their responsiveness.

### Behavioural analysis

Prior to analysis, the videos of the trials were edited in iMovie (Apple Inc.) so that each file presented a single trial with a sound effect replacing the target phrase. All videos were blind coded in Sportscode Gamebreaker 11 (Sportstec, Warriewood, NSW, Australia) by HRG and 25% were second-coded by ATK. Response was defined as the dog directing its gaze towards the owner. The binary gaze response following presentation of the target phrase and duration of response were recorded for each trial. The duration of reaction was capped at 10 s, which was the maximum length of speech measured from the start of the target phrase to the end of the extract. Inter-observer rating agreement was measured for binary gaze and duration using Cronbach’s alpha. This resulted in a score of 0.98 out of 1 for binary gaze and 0.94 out of 1 for duration of gaze, which is considered excellent (Bland and Altman 1997).

All coding data files are available as Excel files in the electronic supplementary material and available on Dryad (10.5061/dryad.stqjq2c1s).

### Statistics

All statistics were performed using SPSS (SPSS Inc., Chicago, IL., USA) v. 25 and v. 27. Binomial generalized linear mixed effect models (GLMMs) were applied to the binary gaze response variable to examine the effect of tone and content on gaze response. Duration of response was measured in trials where dogs demonstrated a gaze response, and normal-distribution linear mixed effect models (LMMs) fitted with restricted-maximum likelihood estimation were used to examine the effect of tone and content on gaze duration. For all models, dog identity was included as a random effect and fixed effects were meaning & speech register (DDS or NRP), dog age, and sex. In Experiments 1 and 2, speaker sex was also included as a fixed effect. For experiment 2: Sex, the trial number was included as a random effect. Data were checked for violations of GLMM and LMM assumptions and were not found to be in violation, with residuals normally distributed for the LMM. Bonferroni corrections were used for the p-values throughout, set according to the number of comparisons. Only full models were considered with no stepwise selection undertaken.

## Results and discussion

Full results of all models below are presented in Electronic Supplementary Tables 3–8.

### Pilot: the effect of meaning on dogs’ responses to content presented in dog-directed speech (DDS) prosody

**Fig. 2:**
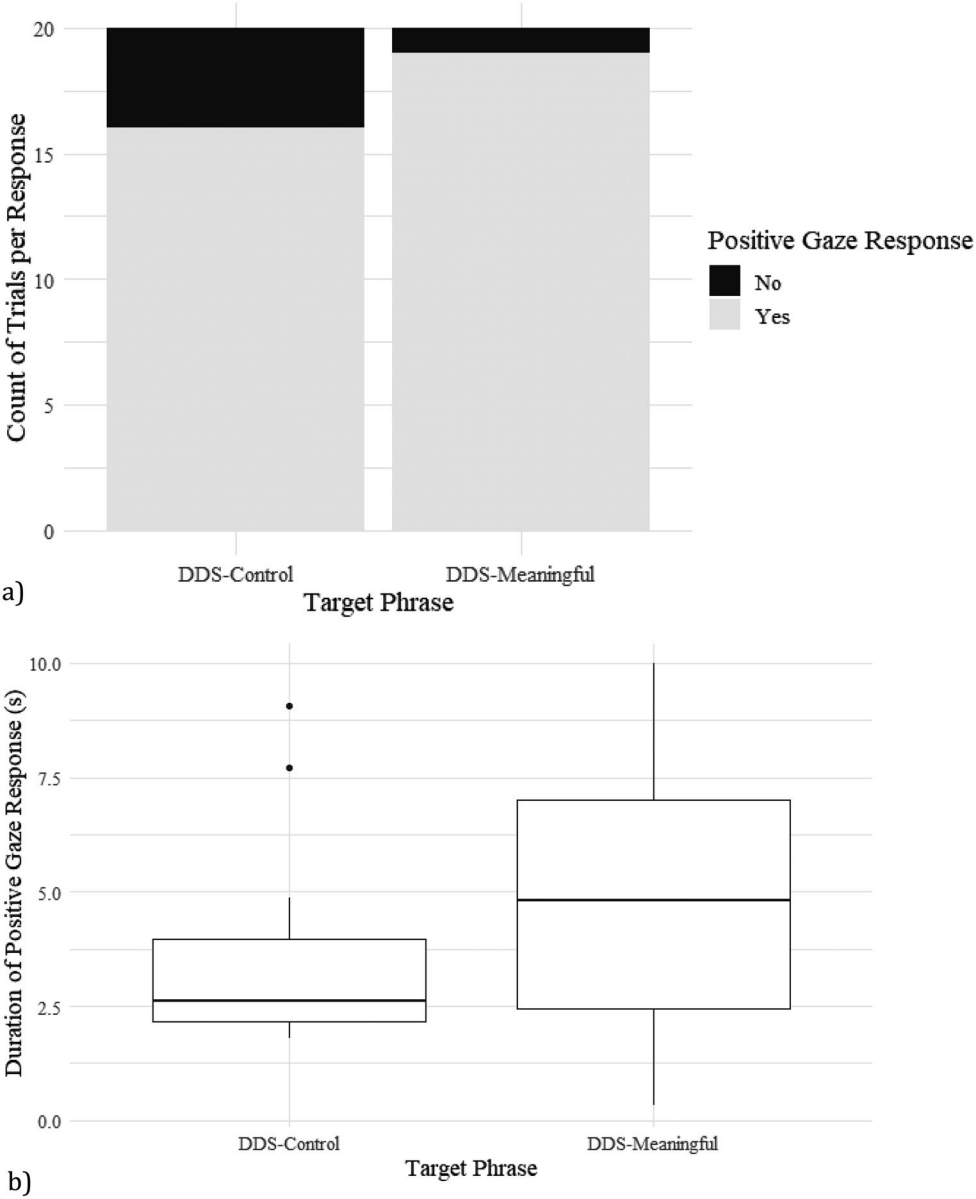
Pilot Experiment: **a**) Stacked bar chart of binary gaze towards owner by dogs in response to target phrases spoken in DDS. Target phrase did not have a significant effect on binary gaze response (F_1,29_ = 3.0, *p* = 0.096). **b**) Boxplot of duration of gaze response to the phrases, where target phrase had no significant effect (F_1,16_ = 3.3, *p* = 0.087)

In the pilot experiment, where only DDS phrases were presented, the dogs reacted to the phrase inclusions in 87.5% of trials (35 out of 40), only showing no response in 4 DDS-control trials and 1 DDS-meaningful trial (Table 2; Fig. 2a). (NB: The one dog that did not respond in either trial showed no obvious signs of hearing impediment and responded readily to his name outside of the experiment.) The binary gaze response variable was not significantly affected by speech content (binomial GLMM: F_1,36_ = 2.1, *p* = 0.155), dog age (F_8,29_ = 0.0, *p* = 1.000), or dog sex (F_1,29_ = 0.3, *p* = 0.601).

The LMM revealed no significant effect of speech content (F_1,16_ = 3.3, *p* = 0.087), dog age (F_8,9_ = 1.2, *p* = 0.412) or sex (F_1,8_ = 1.0, *p* = 0.347) on duration of positive gaze response once the response was elicited (Fig. 2b). 95% confidence intervals for DDS-control were lower bound = 2.7s and upper bound = 5.5s, vs. for DDS-meaningful 4.3s and 6.8s respectively, mean difference = 1.5s.

It was concluded that dogs responded to DDS without other cues from their owner when played from a speaker but that this was not influenced by speech content. Therefore, further tests of responsiveness to speech were undertaken.

**Table 2:** Count of trials with positive gaze at owner and the mean and standard deviation of duration of positive gaze at owner response for each experiment by phrase, with only positive responses included for gaze duration

Study	Variable	NRP-control	NRP-meaningful	DDS-control	DDS-meaningful
Pilot	N of positive gaze / total trials	-	-	16 / 20	19 / 20
	Duration (s) Std. Dev.	-	-	3.49 (2.13)	5.11 (2.98)
1	N of positive gaze / total trials	5 / 43	26 / 43	26 / 43	42 / 43
	N of dogs showing positive gaze / total dogs tested	5 / 31	26 / 31	20 / 31	30 / 31
	Duration (s) Std. Dev.	1.72 (1.1)	2.37 (1.1)	3.09 (2.2)	3.92 (2.5)
2	N of positive gaze / total trials	2 / 24	15/ 24	17 / 24	24 / 24
	N of dogs showing positive gaze / total dogs tested	2/12	10 / 12*	11 / 12**	12 / 12
	Duration (s) Std. Dev.	1.06 (2.0)	2.46 (1.3)	2.68 (1.6)	3.35 (2.2)

NB: In Experiment 2 Sex, the dogs were given 2 opportunities to respond to the sound of their owner’s voice in each condition. Thus, we noted whether they responded differently to male and female owners thus

* 2 dogs did not respond to the NRP-meaningful phrase from either owner, 2 responded only to the female owner and 3 only to the male owner

**1 dog did not respond to the DDS-control phrase from either owner, 2 responded only to the female owner and 3 only to the male owner

### Experiment 1 prosody: effects of prosody and meaningful content on response

**Fig. 3:**
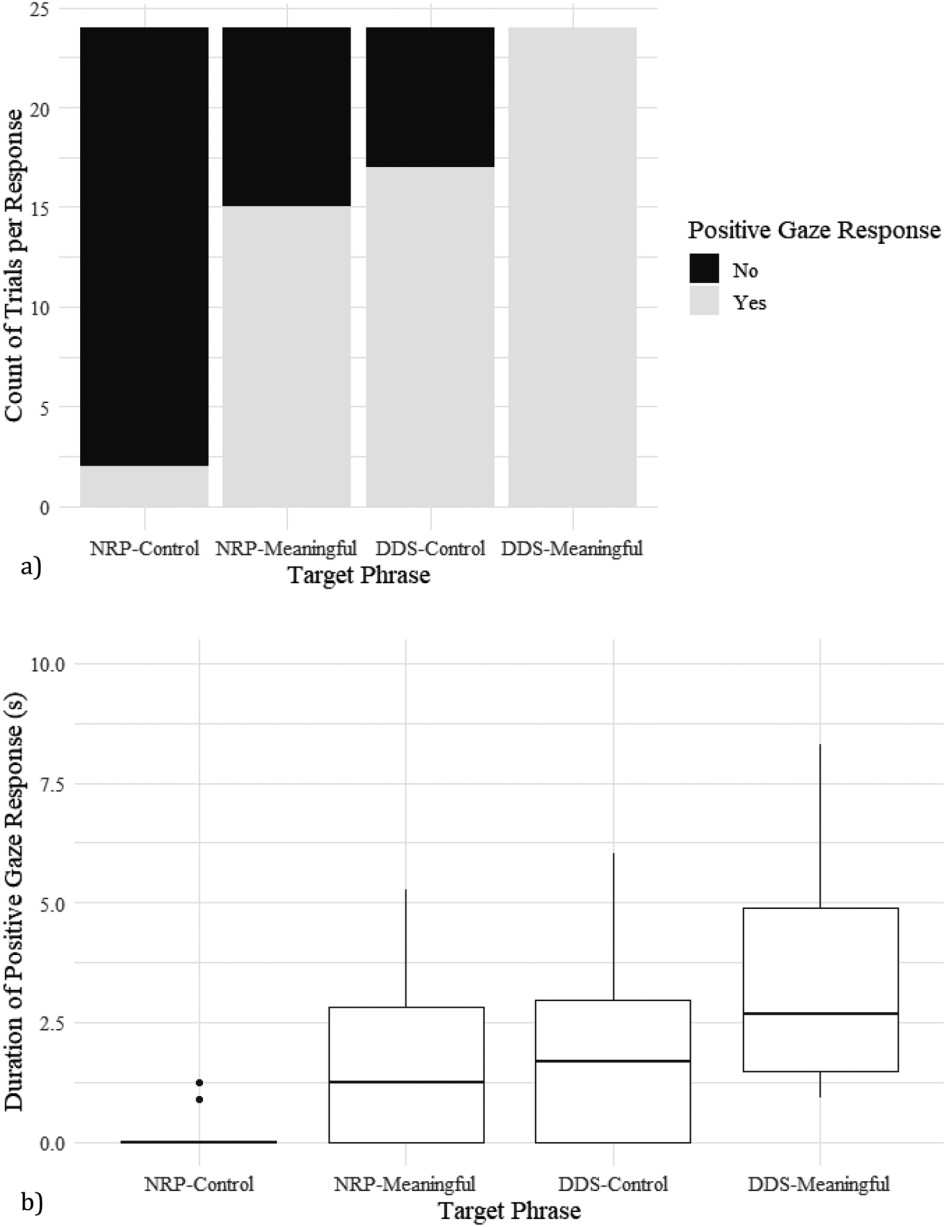
**a**) Stacked bar chart plot of binary gaze towards owner by dogs in response to target phrases spoken in: (1) neutral reading prosody (NRP)-control phrase, (2) NRP-meaningful phrase, (3) dog-directed prosody (DDS)-control phrase, and (4) DDS-meaningful phrase. Target phrase had a significant effect on binary gaze response (F_3,165_ = 12.3, *p* < 0.001), with DDS-meaningful and NRP-Control differing from all other conditions, while NRP-meaningful did not differ to DDS-Control (*p* = 0.100). **b**) Boxplot of the duration of gaze (s) in positive reactions to each phrase. Gaze duration of positive reactions did not differ across phrases except for DDS-meaningful to all others (*p* < 0.05) but was significant overall (F_3,92_ = 4.8, *p* = 0.004, *p* < 0.017 Bonferroni correction value)

For Experiment 1: Prosody, where we examined the dogs’ overall responses to prosody and meaningful content, the dogs showed a positive gaze response to their owners in 57.6% of trials (99 of 172, Table 2). Due to multiple comparisons, we used a Bonferroni correction and significance was *p* < 0.017. The GLMM showed that there was a significant effect of speech on the binary gaze response (F_3,165_ = 12.3, *p* < 0.001), but there was no significant effect of owner sex (F_1,165_ = 4.0, *p* = 0.046), dog age (F_1,165_ = 0.7, *p* = 0.390), or dog sex (F_1,165_ = 1.2, *p* = 0.272). Post-hoc tests showed that all pairwise comparisons of reactions to the four phrase-types were significant at *p* < 0.001 except between NRP-meaningful and DDS-control (*p* = 1.000, see ESM Table 5 for full pairwise comparisons). Overall, dogs were most likely to look at their owners in the DDS-meaningful condition, and least likely in the NRP-control condition (Fig. 3a).

The LMM showed that duration of gaze in trials when the dog chose to gaze at their owner was significantly different by phrase (F_3,92_ = 4.8, *p* = 0.004, Fig. 3b), but not other factors (dog age: F_1,92_ = 0.3, *p* = 0.583; Owner sex F_1,92_ = 0.3, *p* = 0.599, male owner 95% confidence intervals (CI): 1.8–3.5 s vs. female owner CI: 2.1–3.6 s; dog sex F_1,92_ = 0.7, *p* = 0.417, male dog CI: 2.1–3.9 s vs. female dog CI: 1.6–3.4 s). The target phrase DDS-meaningful (CI: 3.2–4.7 s) differed from all other phrases: NRP-control (CI: 2.1–3.9 s, *p* = 0.002), NRP-meaningful (CI: 1.6–3.3 s, *p* = 0.014), and DDS-control (CI: 2.1–3.9 s, *p* = 0.046). All other comparisons between DDS-control, NRP-control, and NRP-meaningful were not significant at *p* < 0.05 (see ESM for full comparisons).

### Experiment 2 sex: effects of speaker sex on response to prosody and meaningful content

**Fig. 4:**
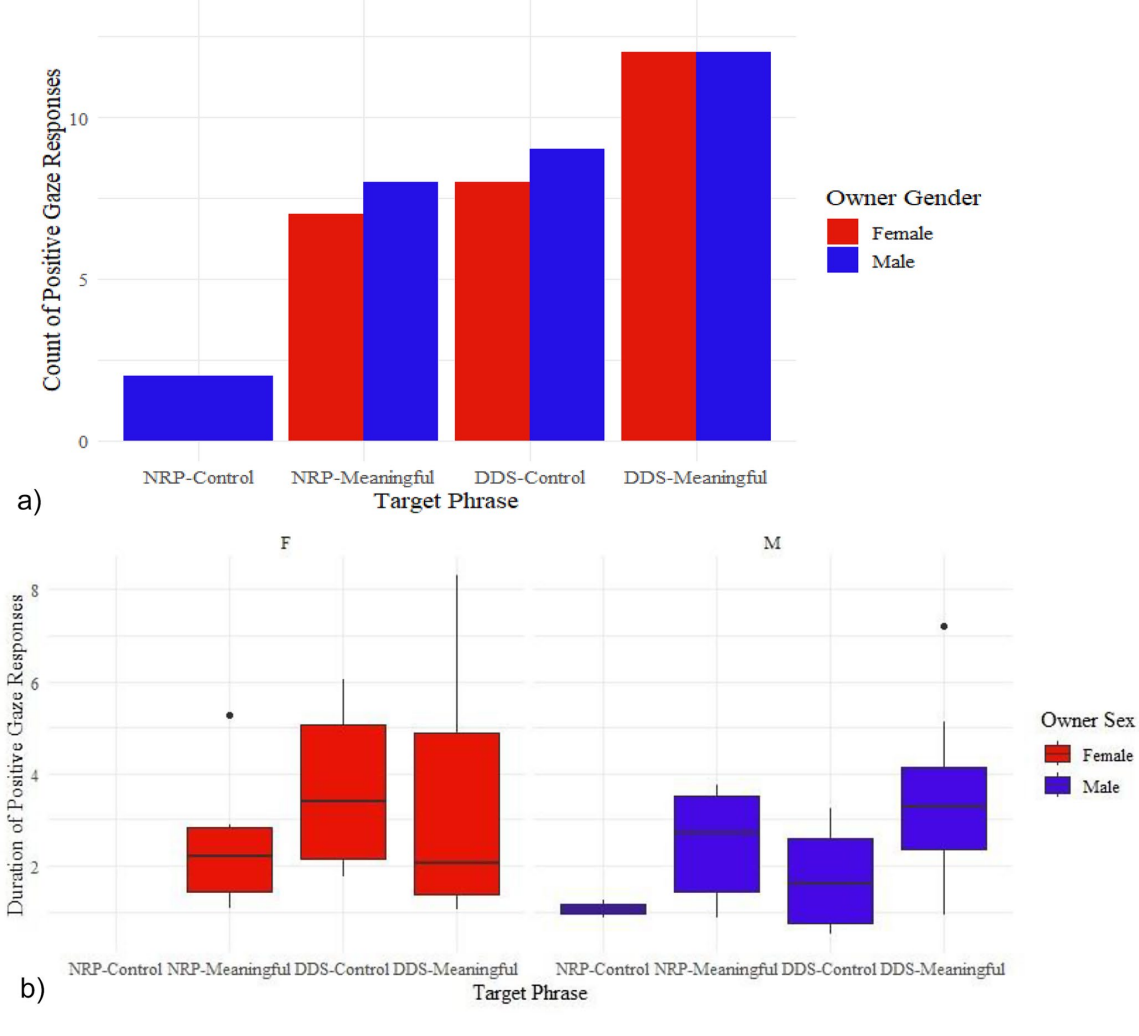
**a**) Stacked bar chart plot of binary gaze towards owner by dogs in response to target phrases spoken in: (1) neutral reading prosody (NRP)-control phrase, (2) NRP-meaningful phrase, (3) dog-directed prosody (DDS)-control phrase, and (4) DDS-meaningful phrase. Target phrase had a significant effect on binary gaze response (F_3,89_ = 5.4, *p* = 0.002), with DDS-meaningful and NRP-Control differing from all other conditions, while NRP-meaningful did not differ to DDS-Control. **B**) Boxplot of the duration of gaze (s) in positive reactions to each phrase, grouped by owner sex. Gaze duration of positive reactions did not differ across phrases or with owner sex at *p* < 0.017. (Note: No dog responded to NRP-control from their female owner, thus NRP-Control positive gaze duration is empty)

For Experiment 2 Sex, where we examined the effect of speaker sex on dogs’ responses, dogs showed a positive gaze response to their owner in 60.4% of trials (58 of 96, Fig. 4a). The GLMM showed that there was a significant effect of phrase on the binary gaze response (F_3,89_ = 5.4, *p* = 0.002), but owner sex (F_1,89_ = 1.4, *p* = 0.239), dog age (F_1,89_ = 1.6, *p* = 0.211), or dog sex (F_1,89_ = 0.5, *p* = 0.495) had no significant effect. Post-hoc tests showed that all pairwise comparisons of reactions to the four phrase-types were significant at *p* < 0.017 except between NRP-meaningful and DDS-control (*p* = 0.517, see ESM Table 7 for all post-hoc comparisons). Overall, dogs were most likely to look at their owners in the DDS-meaningful condition, and least likely in the NRP-control condition (Fig. 4a).

The LMM showed that duration of gaze in trials when the dog chose to gaze at their owner did not differ significantly between any fixed effects (*p* > 0.3 for all, see ESM Table 8 for full details, Fig. 4b).

## Discussion

We found that dogs spontaneously recognise meaningful phonemic content presented within streams of putatively non-salient speech. Dogs were more likely to look at their owner when the meaningful phrase was presented in dog directed speech (DDS), but they also detected the meaningful phrase when presented in neutral reading prosody (NRP) more often than the control phrase. Thus, dogs’ ability to recognise familiar phrase within a stream of speech was not systematically conditional on specific exaggerated prosodic guidance, though this may have been aided by pauses. We support previous results in both dogs (Ratcliffe and Reby 2014; Benjamin and Slocombe 2018) and human infants (Thiessen et al. 2005) which indicated that exaggerated prosodic cues increase attention. Here, we show that while DDS is sufficient to engage dogs’ attention without meaningful content, as the dogs responded to the control phrase in DDS at equal rates to the meaningful phrase in NRP speech, content is also important as the dogs responded more often to the combined presentation of DDS with meaningful content and still responded to the presentation of meaningful content presented in NRP. These observations add weight to the idea that dogs separate the speech signals into phonemic and prosodic elements and can respond to both independently (Scheider et al. 2011; Reinholz-Trojan et al. 2012; Andics et al. 2014; Ratcliffe and Reby 2014; Gábor et al. 2020).

When we analysed trials where dogs responded by gazing at their owners, we found that gaze duration was only affected in Experiment 1 and not in Experiment 2. This lack of differences found in Experiment 2 is unlikely to be caused by a ceiling effect as the dogs were given a maximum of 10 s to respond from onset of the included phrase, and no dogs reached this ceiling in Experiments 1 or 2. This may also be a consequence of the small number of dogs that responded to NRP-control presentations (just 4 out of 25 in Experiment 1). Because we were not able to replicate the differences in gaze duration, we consider the finding tentative.

Women produce more exaggerated DDS speech compared to their NRP speech than men. Men also use less DDS prosody when talking to their dog (Prato-Previde et al. 2006), potentially making dogs more familiar with male NRP speech directed at them. Thus, we had predicted that female DDS-speech would be more attention-invoking than male DDS speech, and that male NRP speech would elicit a greater response than female NRP speech. These predictions were not confirmed by our data. Instead, we found that dogs did not respond more readily to presentations of male NRP or female DDS speech, and sex did not influence dogs’ responses to speech content. Indeed, we found no significant effect of sex on the dogs’ overall responsiveness or duration of response. Thus, despite differences in how men and women use speech to dogs, dogs showed similar attention to speech prosody across the sexes. We did find a potential effect of dogs’ sex on duration of positive gaze response, but the sample size for this was not sufficient to be conclusive and this result should be considered tentative.

In a pet dog’s typical environment, most human speech is likely directed towards humans. As such, DDS has been posited to function as a communication strategy aimed at signalling to the dog that the speech is intended for them (Ben-Aderet et al. 2017; Benjamin and Slocombe 2018), and that highly modulated pitch and increased range are attention-getting devices (Lesch et al. 2019). One may thus hypothesise that dogs would require such prosody in order to detect meaningful verbal signals and fail to respond to meaningful content “hidden” (Kaminski et al. 2012) within streams of non-exaggerated speech. However, we found that dogs still have the ability to parse speech in the absence of exaggerated prosodic cueing, an ability that has been hypothesised to be uniquely human and specific to speech perception (Mandel et al. 1995; Reinholz-Trojan et al. 2012). Since speaker voice familiarity has been shown to influence speech perception in human infants (Barker and Newman 2004; Naoi et al. 2012; Trehub 2017), further research could explore whether voice familiarity also affects speech perception in dogs. As it is known that familiar word position within sentences influence infants’ ability to segment speech (Seidl and Johnson 2006), testing dogs’ responses to a more varied range of target cues, for example embedding their name within a phrase as Floccia et al. (2016) did, would allow direct comparisons with human abilities.

Our behavioural observations lend support to previous indications that non-human animals are capable of segmenting human speech (Hauser et al. 2001; Toro and Trobalón 2005; Lu and Vicario 2014), including Boros et al.’s (2021) report that dogs possess the neural abilities required for speech segmentation. We suggest that the ability to parse complex vocal utterances is not specific to humans. Furthermore, it may be linked to the ability to recognise alarm call units which can be presented against noise, overlapping calls, or within longer sequences, by conspecifics or heterospecifics. The ability to parse complex call sequences to recognise familiar, salient units is important to vocal learning in general (Elowson et al. 1998; Clay and Zuberbühler 2011) as well as heterospecific call recognition (Morris-Drake et al. 2017; Zhou et al. 2024).

One limitation to our study is the fact that no dogs were present in the room when the dog owners’ speech was recorded. This was deliberate in order to avoid the dogs habituating to the speech. Instead, we asked dog owners to imagine they were talking to their dogs. This less-than-realistic set up may have affected some features of DDS in dog owners’ speech (Ben-Aderet et al. 2017). In order to improve the ecological validity of the study (Jeannin et al. 2017) in future experiments dogs could be present during the recording of their owners’ DDS. However, we did find the differences in NRP and DDS to be in line with what would be expected for the different speech registers (Burnham et al. 2002).

A further limitation is that speech segmentation studies usually present the speech without including a brief pause before the target phrase. Our speakers produced natural pauses of 0.05–0.6 s, with an average of 0.4s, as a comma was presented before the included phrases. These brief, natural pauses may have helped to cue the dogs to the presence of a new phrase and thus increased their attention to it. However, they were present before all included phrases, as well as at other points in the text, so while they may have increased attention and thus meaningful phrase recognition, we do not believe that they can account for the responses. Further studies specifically presenting the included phrase embedded without a natural pause (and thus coarticulated with surrounding speech) would allow an estimate of the effect of natural pauses in our experiment, as well as further elucidate the extent to which dog speech parsing abilities compare to that of humans.

Our finding is consistent with previous observations that domestic dogs respond to the phonemic content of short speech signals in the absence of exaggerated prosody(Ratcliffe and Reby 2014) and that they use specific brain regions for processing the verbal and nonverbal content of human speech (Andics et al. 2014; Andics and Miklósi 2018; Gábor et al. 2020; Boros et al. 2020). This ability may reflect the effect of selective breeding on dogs to respond to human vocal signals (Hare et al. 2002). To further investigate this, experiments could extend this study to tame wolves (*Canis lupus*), an undomesticated close relative of dogs, as well as to other domesticated species that are regularly exposed to human speech, e.g., cats and horses which have been shown to be sensitive to various aspects of the human voice and speech (Proops et al. 2012; Saito and Shinozuka 2013; Heleski et al. 2015; Galvan and Vonk 2016; Nakamura et al. 2018; Takagi et al. 2019; Saito et al. 2019).

## Electronic supplementary material

Below is the link to the electronic supplementary material.

Supplementary Material 1

Supplementary Material 2

Supplementary Material 3

Supplementary Material 4

Supplementary Material 5

Supplementary Material 6

Supplementary Material 7

Supplementary Material 8

## Data Availability

All coding data files are available as Excel files in the electronic supplementary material and the original videos are available on Dryad (DOI: 10.5061/dryad.stqjq2c1s).
