# Supplementary material for: Domestic dogs (Canis familiaris) recognise meaningful content in monotonous streams of read speech
Source: Anim Cogn. 2025 Apr 12;28(1):29. doi: 10.1007/s10071-025-01948-z (PMC11993455; doi:10.1007/s10071-025-01948-z)
Supplement: Supplementary file 1 — Supplementary Material 1 [file 10071_2025_1948_MOESM1_ESM.docx]

**Table 5: Results for GLMM post-hoc comparisons of phrase, Study 1.**

| **Pairwise Contrasts** | | | | | | | |
| --- | --- | --- | --- | --- | --- | --- | --- |
| Target Phrase Pairwise Contrasts | Contrast Estimate | Std. Error | t | df | Adj. Sig. | 95% Confidence Interval | |
|  |  |  |  |  |  | Lower | Upper |
| **DDS-Control - DDS-Meaningful** | **.325** | **.083** | **3.893** | **165** | **<0.001** | **.160** | **.489** |
| **DDS-Control - NRP-Control** | **-.544** | **.095** | **-5.748** | **165** | **<0.001** | **-.731** | **-.357** |
| DDS-Control - NRP-Meaningful | .000 | .104 | .000 | 165 | 1.000 | -.206 | .206 |
| **DDS-Meaningful - DDS-Control** | **-.325** | **.083** | **-3.893** | **165** | **<0.001** | **-.489** | **-.160** |
| **DDS-Meaningful - NRP-Control** | **-.869** | **.054** | **-16.026** | **165** | **<0.001** | **-.976** | **-.762** |
| **DDS-Meaningful - NRP-Meaningful** | **-.325** | **.083** | **-3.893** | **165** | **<0.001** | **-.489** | **-.160** |
| **NRP-Control - DDS-Control** | **.544** | **.095** | **5.748** | **165** | **<0.001** | **.357** | **.731** |
| **NRP-Control - DDS-Meaningful** | **.869** | **.054** | **16.026** | **165** | **<0.001** | **.762** | **.976** |
| **NRP-Control - NRP-Meaningful** | **.544** | **.095** | **5.748** | **165** | **<0.001** | **.357** | **.731** |
| NRP-Meaningful - DDS-Control | .000 | .104 | .000 | 165 | 1.000 | -.206 | .206 |
| **NRP-Meaningful - DDS-Meaningful** | **.325** | **.083** | **3.893** | **165** | **<0.001** | **.160** | **.489** |
| **NRP-Meaningful - NRP-Control** | **-.544** | **.095** | **-5.748** | **165** | **<0.001** | **-.731** | **-.357** |
| The least significant difference adjusted significance level is .05. | | | | | | | |
