# Supplementary material for: Domestic dogs (Canis familiaris) recognise meaningful content in monotonous streams of read speech
Source: Anim Cogn. 2025 Apr 12;28(1):29. doi: 10.1007/s10071-025-01948-z (PMC11993455; doi:10.1007/s10071-025-01948-z)
Supplement: Supplementary file 2 — Supplementary Material 2 [file 10071_2025_1948_MOESM2_ESM.docx]

**Table 6: Post-hoc comparisons of target phrase in LMM, Study 1. Significance marked in bold.**

| **Pairwise Comparisons** | | | | | | | |
| --- | --- | --- | --- | --- | --- | --- | --- |
| (I) Target Phrase | (J) Target Phrase | Mean Difference (I-J) | Std. Error | df | Sig. | 95% Confidence Interval for Difference | |
|  |  |  |  |  |  | Lower Bound | Upper Bound |
| DDS-Control | **DDS-Meaningful** | **-.968^*^** | **.478** | **70.812** | **.047** | **-1.922** | **-.014** |
|  | NRP-Control | 1.455 | .995 | 80.566 | .147 | -.525 | 3.436 |
|  | NRP-Meaningful | .556 | .535 | 72.033 | .302 | -.511 | 1.623 |
| DDS-Meaningful | **DDS-Control** | **.968^*^** | **.478** | **70.812** | **.047** | **.014** | **1.922** |
|  | **NRP-Control** | **2.423^*^** | **.963** | **79.431** | **.014** | **.507** | **4.340** |
|  | **NRP-Meaningful** | **1.524^*^** | **.480** | **70.434** | **.002** | **.567** | **2.482** |
| NRP-Control | DDS-Control | -1.455 | .995 | 80.566 | .147 | -3.436 | .525 |
|  | **DDS-Meaningful** | **-2.423^*^** | **.963** | **79.431** | **.014** | **-4.340** | **-.507** |
|  | NRP-Meaningful | -.899 | .991 | 78.823 | .367 | -2.871 | 1.073 |
| NRP-Meaningful | DDS-Control | -.556 | .535 | 72.033 | .302 | -1.623 | .511 |
|  | **DDS-Meaningful** | **-1.524^*^** | **.480** | **70.434** | **.002** | **-2.482** | **-.567** |
|  | NRP-Control | .899 | .991 | 78.823 | .367 | -1.073 | 2.871 |
