# Supplementary material for: Domestic dogs (Canis familiaris) recognise meaningful content in monotonous streams of read speech
Source: Anim Cogn. 2025 Apr 12;28(1):29. doi: 10.1007/s10071-025-01948-z (PMC11993455; doi:10.1007/s10071-025-01948-z)
Supplement: Supplementary file 4 — Supplementary Material 4 [file 10071_2025_1948_MOESM4_ESM.docx]

**Table 7: Post-hoc comparisons for GLMM of target phrase, Study 2. Significant results at p < 0.017 are marked in bold.**

| **Pairwise Contrasts** | | | | | | | |
| --- | --- | --- | --- | --- | --- | --- | --- |
| Target Phrase Pairwise Contrasts | Contrast Estimate | Std. Error | t | df | Adj. Sig. | 95% Confidence Interval | |
|  |  |  |  |  |  | Lower | Upper |
| **DDS-Control - DDS-Meaningful** | **.283** | **.113** | **2.500** | **89** | **.014** | **.058** | **.508** |
| **DDS-Control - NRP-Control** | **-.658** | **.117** | **-5.628** | **89** | **<0.001** | **-.890** | **-.426** |
| DDS-Control - NRP-Meaningful | -.096 | .147 | -.651 | 89 | .517 | -.388 | .197 |
| **DDS-Meaningful - DDS-Control** | **-.283** | **.113** | **-2.500** | **89** | **.014** | **-.508** | **-.058** |
| **DDS-Meaningful - NRP-Control** | **-.941** | **.046** | **-20.344** | **89** | **<0.001** | **-1.033** | **-.849** |
| **DDS-Meaningful - NRP-Meaningful** | **-.379** | **.126** | **-3.015** | **89** | **.003** | **-.628** | **-.129** |
| **NRP-Control - DDS-Control** | **.658** | **.117** | **5.628** | **89** | **<0.001** | **.426** | **.890** |
| **NRP-Control - DDS-Meaningful** | **.941** | **.046** | **20.344** | **89** | **<0.001** | **.849** | **1.033** |
| **NRP-Control - NRP-Meaningful** | **.562** | **.127** | **4.415** | **89** | **<0.001** | **.309** | **.815** |
| NRP-Meaningful - DDS-Control | .096 | .147 | .651 | 89 | .517 | -.197 | .388 |
| **NRP-Meaningful - DDS-Meaningful** | **.379** | **.126** | **3.015** | **89** | **.003** | **.129** | **.628** |
| **NRP-Meaningful - NRP-Control** | **-.562** | **.127** | **-4.415** | **89** | **<0.001** | **-.815** | **-.309** |
| The least significant difference adjusted significance level is .05. | | | | | | | |
