# Supplementary material for: Domestic dogs (Canis familiaris) recognise meaningful content in monotonous streams of read speech
Source: Anim Cogn. 2025 Apr 12;28(1):29. doi: 10.1007/s10071-025-01948-z (PMC11993455; doi:10.1007/s10071-025-01948-z)
Supplement: Supplementary file 5 — Supplementary Material 5 [file 10071_2025_1948_MOESM5_ESM.docx]

**Table 8: Full LMM results for study 2. No fixed effects were significant at p <0.05.**

| **Type III Tests of Fixed Effects** | | | | |
| --- | --- | --- | --- | --- |
| Source | Numerator df | Denominator df | F | Sig. |
| Intercept | 1 | 5.098 | 21.847 | .005 |
| Age | 7 | 2.118 | .254 | .930 |
| Sex | 1 | 3.050 | .006 | .944 |
| Target Phrase | 3 | 42.328 | 1.231 | .310 |
| Owner Gender | 1 | 42.445 | .609 | .440 |
|  | | | | |
