# Supplementary material for: Domestic dogs (Canis familiaris) recognise meaningful content in monotonous streams of read speech
Source: Anim Cogn. 2025 Apr 12;28(1):29. doi: 10.1007/s10071-025-01948-z (PMC11993455; doi:10.1007/s10071-025-01948-z)
Supplement: Supplementary file 6 — Supplementary Material 6 [file 10071_2025_1948_MOESM6_ESM.docx]

**Table 4: LMM results for Pilot Study, 20 dogs. This only included female owners.**

| **Fixed effect** | **F** | **df1** | **df2** | **p-value** |
| --- | --- | --- | --- | --- |
| Intercept | 1.222 | 2 | 29 | 0.308 |
| Age | 0.280 | 1 | 29 | 0.601 |
| Phrase | 2.961 | 1 | 29 | 0.096 |
| Sex | 0.025 | 1 | 29 | 1.000 |
